# Super-resolution imaging reveals nucleolar encapsulation by single-stranded DNA

**DOI:** 10.1242/jcs.262039

**Published:** 2024-10-04

**Authors:** Koichiro Maki, Jumpei Fukute, Taiji Adachi

**Affiliations:** ^1^ Laboratory of Biomechanics, Institute for Life and Medical Sciences, Kyoto University, 53 Shogoin-Kawahara, Sakyo, Kyoto 606-8507, Japan; ^2^ Department of Micro Engineering, Graduate School of Engineering, Kyoto University, 53 Shogoin-Kawahara, Sakyo, Kyoto 606-8507, Japan; ^3^ Department of Mammalian Regulatory Network, Graduate School of Biostudies, Kyoto University, 53 Shogoin-Kawahara, Sakyo, Kyoto 606-8507, Japan; ^4^ Department of Medicine and Medical Science, Graduate School of Medicine, Kyoto University, 53 Shogoin-Kawahara, Sakyo, Kyoto 606-8507, Japan

**Keywords:** Single-stranded DNA, Nucleolus, Nucleus, DNA–protein interaction, *In situ* imaging, Super-resolution imaging

## Abstract

In eukaryotic cell nuclei, specific sets of proteins gather in nuclear bodies and facilitate distinct genomic processes. The nucleolus, a nuclear body, functions as a factory for ribosome biogenesis by accumulating constitutive proteins, such as RNA polymerase I and nucleophosmin 1 (NPM1). Although *in vitro* assays have suggested the importance of liquid–liquid phase separation (LLPS) of constitutive proteins in nucleolar formation, how the nucleolus is structurally maintained with the intranuclear architecture remains unknown. This study revealed that the nucleolus is encapsulated by a single-stranded (ss)DNA-based molecular complex inside the cell nucleus. Super-resolution lattice-structured illumination microscopy (lattice-SIM) showed that there was a high abundance of ssDNA beyond the ‘outer shell’ of the nucleolus. Nucleolar disruption and the release of NPM1 were caused by *in situ* digestion of ssDNA, suggesting that ssDNA has a structural role in nucleolar encapsulation. Furthermore, we identified that ssDNA forms a molecular complex with histone H1 for nucleolar encapsulation. Thus, this study illustrates how an ssDNA-based molecular complex upholds the structural integrity of nuclear bodies to coordinate genomic processes such as gene transcription and replication.

## Teaser

**Summary:** Super-resolution imaging reveals that the cell nucleolus is encapsulated by molecular complex of single-stranded DNA and histone H1.

## INTRODUCTION

In eukaryotic cells, the nucleus contains membrane-less structures called nuclear bodies (Lanctôt et al., 2007; Mao et al., 2011; Staněk and Fox, 2017). Nuclear bodies comprise sets of proteins that drive specific genomic processes (Banani et al., 2016; Blocher McTigue and Perry, 2020; Meldi and Brickner, 2011). For example, the nucleolus serves as a factory for ribosome biogenesis by accumulating constitutive proteins, such as RNA polymerase I (RNAPI), fibrillarin (FIB, also known as FBL) and nucleophosmin 1 (NPM1) (Frottin et al., 2019; Lafontaine et al., 2021). The nucleolus exhibits a ‘core–shell’ architecture, where ribosomal RNA (rRNA) is transcribed, processed and modified in the ‘core’, and ribonucleoprotein is assembled, transported and quality-controlled in the ‘shell’. *In vitro* assays have suggested that liquid–liquid phase separation (LLPS) of constitutive proteins is important in the core–shell architecture of nucleolus (Banani et al., 2016; Feric et al., 2016). Importantly, the nucleolus is associated with intranuclear components, and some studies have suggested that intranuclear architectures, such as filamentous actin, regulate the size and morphology of the nucleolus (Feric and Brangwynne, 2013; Talbot et al., 2023). Thus, it is important to consider the structural roles of intranuclear components in the nucleolar architecture.

In this study, as one of the intranuclear components that contributes structurally to the nucleolar architecture, we focused on the single-stranded (ss)DNA-based molecular complex. Gene replication and transcription are initiated by the separation of double-stranded (ds)DNA into single-stranded DNAs (Patel and Donmez, 2006; van Brabant et al., 2000), with which DNA and RNA polymerases, and transcription factors are associated. Besides its role as a substrate for various genomic processes, such as replication, transcription and repair, ssDNA plays a structural role by forming biomolecular condensates. *In vitro* reconstitution experiments have reported that ssDNA forms condensates by interacting with various binding proteins (Harami et al., 2020; Renger et al., 2022; Stender et al., 2021). Another study has reported that ssDNA forms a denser condensate with histone H1 than it does with dsDNA, suggesting that it has a possible structural role in the nucleus (Leicher et al., 2022; Nott et al., 2016). Therefore, we hypothesized that ssDNA contributes to the nucleolar architecture by forming a molecular complex with a binding protein.

To explore the structural roles of ssDNA in the nucleolar architecture, it is necessary to identify the distribution of ssDNA associated with the nucleolus. Thus, *in situ* imaging is a powerful approach. Multiple trials have been conducted to visualize ssDNA inside the nucleus. For example, immunofluorescence staining for replication protein A (RPA) and Rad51 (Raderschall et al., 1999), which are the major ssDNA-binding proteins, has been used to label ssDNA. Furthermore, by taking advantage of ssDNA to form secondary structures, immunofluorescence (Moye et al., 2015) and chemical labeling for the G-quadruplex (G4) (Summers et al., 2021), a secondary structure of ssDNA, have also been used. However, direct *in situ* imaging of ssDNA itself has not been proposed yet.

Thus, we aimed to understand how ssDNA contributes structurally to the nucleolar architecture. First, *in situ* imaging of ssDNA was performed using a specific fluorescent dye, which was validated by enzymatic digestion and heat denaturation. Second, super-resolution lattice-structured illumination microscopy (lattice-SIM) was performed to identify the intranuclear distribution of ssDNA. Finally, the structural contribution of ssDNA to nucleolar architecture was investigated by *in situ* enzymatic digestion.

## RESULTS

### *In situ* fluorescence imaging of ssDNA in a cell nucleus

**Fig. 1.**
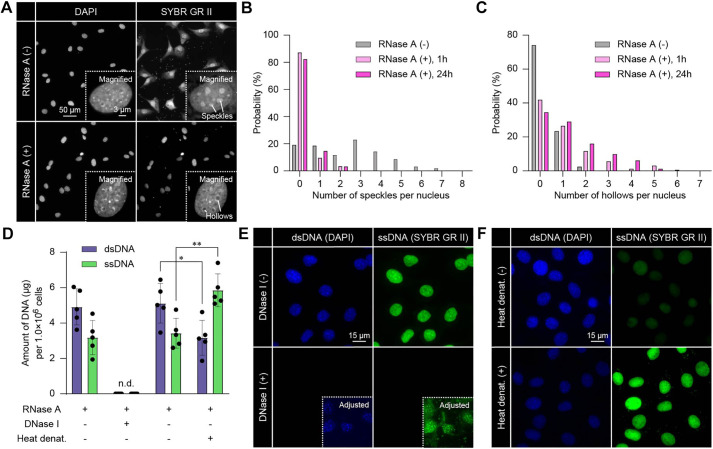
**Verification of *in situ* ssDNA imaging.** (A) Fluorescence images of DAPI (intercalating into dsDNA) and SYBR Green II (binding to both ssDNA and RNA), without and with RNase A treatment. (B,C) Histograms of the number of speckles (B) and hollows (C) per nucleus in the SYBR Green II images. *n*=162 cells [RNase(−)], 147 cells [RNase (+), 1 h], 157 cells [RNase (+), 24 h]. (D) The mean±s.d. amount of dsDNA and ssDNA in cells quantified with the Qubit^TM^ fluorometer. *n*=5 biological replicates. *P<0.05; **P<0.01 (two-sided Mann–Whitney *U* test). n.d., not detected. (E) Fluorescence images without and with DNase I, using semi-intact nuclei. Of note, the exposure time for SYBR Green II was adjusted for heated sample with increased amount of ssDNA, and thereby the signals are relatively weak in the sample without heat denaturation. (F) Fluorescence images without and with heat denaturation. Images in E and F are representative of three repeats.

In this study, we initially validated *in situ* fluorescence imaging of ssDNA using SYBR Green II^TM^ reagent, a commonly used reagent in *in vitro* assays (Saarnio et al., 2020). As SYBR Green II binds to both ssDNA and ribosomal and cytoplasmic RNA, we treated pre-fixed pre-osteoblastic MC3T3-E1 cells with RNase A (Fig. 1A). As a result, RNase A-untreated cells showed speckle-like signals in the nuclei as well as cytoplasmic signals, whereas RNase A-treated cells showed discernible nuclear signals with hollow patterns. Thus, the number of ‘speckles’ and ‘hollows’ per nucleus in SYBR Green II images were decreased and increased by RNase A treatment, respectively (Fig. 1B,C; Fig. S1A). These results are reasonable, as RNA diffuses in entire cells and ribosomal RNA occasionally shows a speckle signal in a nuleolus.

To investigate the binding of SYBR Green II to ssDNA following RNase A treatment, we conducted *in situ* enzymatic digestion and heat denaturation. These procedures were verified with the Qubit^TM^ assay (Fig. 1D). In this experiment, semi-intact nuclei were used for better accessibility of enzymes to the genomic DNA (Nickerson et al., 1997). The digestion of dsDNA and ssDNA with DNase I, followed by RNase treatment, significantly reduced the DAPI and SYBR Green II signals (Fig. 1E). After the heat denaturation of paraformaldehyde (PFA)-fixed cells, a decrease in DAPI signal and a significant increase in SYBR Green II signal were observed (Fig. 1F). These results confirm the specificity of SYBR Green II for *in situ* ssDNA imaging.

### Abundant ssDNA surrounds nucleoli

**Fig. 2.**
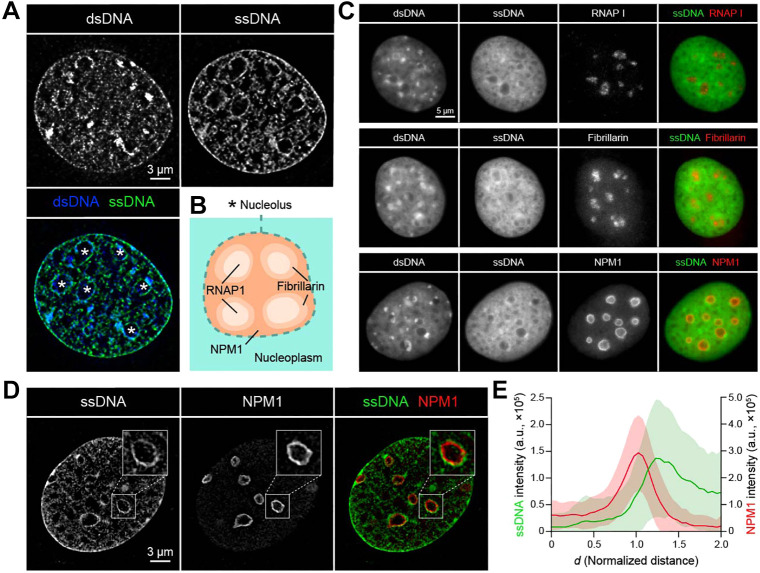
***In situ* super-resolution imaging of ssDNA.** (A) Lattice-SIM images of dsDNA and ssDNA. Hollow-shaped structures are marked with asterisks in the merged image. (B) Schematics of the organization of the nucleolus, comprising three layers: the fibrillar center (FC) containing RNA polymerase I (RNAPI), the dense fibrillar compartment (DFC) containing fibrillarin (FIB), and the granular compartment (GC) containing nucleophosmin 1 (NPM1). (C) Co-staining of dsDNA, ssDNA and nucleolar markers. (D) Lattice-SIM images of ssDNA and NPM1. Images in A, C and D are representative of three repeats. (E) Intensity profile of ssDNA and NPM1. The centroid of nucleolus was determined based on 3D lattice-SIM images of NPM1, and the intensity profiles of ssDNA and NPM1 were measured along the radial axis. The radial distance *d* from the centroid was normalized based on the distance between the centroid and the nucleolar outline. *n*=47 nucleoli from 7 cells. The intensity profile is the moving average with the s.d. indicated as the shaded area. a.u., arbitrary units.

To determine the intranuclear distribution of ssDNA, we conducted super-resolution lattice-structured illumination microscopy (lattice-SIM), which we previously utilized for *in situ* visualization of DNA underwinding (Fukute et al., 2024). The results showed that ssDNA had hollow patterns within cell nuclei (Fig. 2A, asterisks in the bottom left panel). The Qubit^TM^ dye also showed a hollow-shaped distribution of ssDNA (Fig. S1B). The hollow-shaped distribution of intranuclear ssDNA appears to be a general characteristic, as it was observed in mouse embryonic fibroblasts (MEFs) (Fig. S1C). Of note, we observed prominent perinuclear signals, which we discuss in the Discussion section. We also performed immunofluorescence staining for dsDNA and ssDNA (Fig. S1D,E), although we could not detect nuclear localized signals. It is possible that these antibodies detect only cytoplasmic DNA produced upon genotoxic events (Lam et al., 2014; Wu et al., 2020). Co-staining of ssDNA with nucleolar proteins helped to determine in which nucleolar region ssDNA is abundant (Figs. 2B,C). The nucleolus consists of three layers (Fig. 2B): the fibrillar center (FC), which contains RNAPI, the dense fibrillar compartment (DFC), which contains FIB, and the granular compartment (GC), also known as the ‘outer shell’, which contains NPM1 (Frottin et al., 2019; Lafontaine et al., 2021). The staining revealed that ssDNA was abundant outside the nucleolus (Fig. 2C), further confirmed by lattice-SIM (Fig. 2D) and the intensity profile analysis (Fig. 2E). These findings indicate that ssDNA was abundant outside the ‘outer shell’ of the nucleolus.

### Structural role of ssDNA in nucleolar encapsulation

**Fig. 3.**
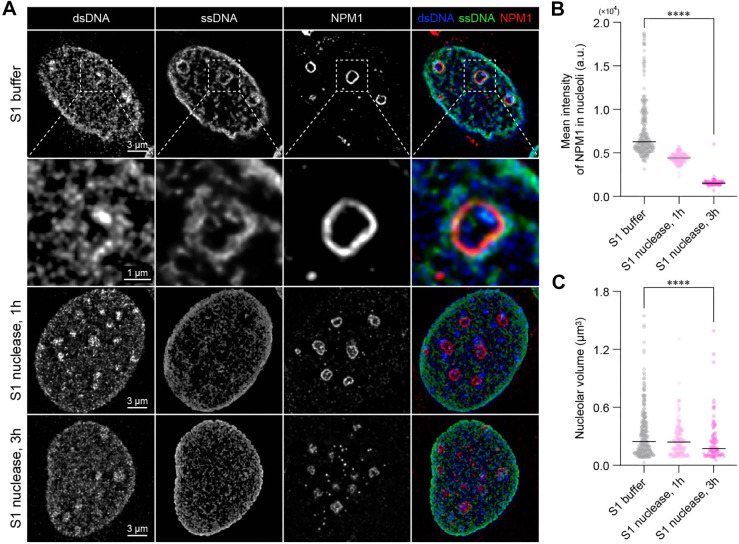
***In situ* digestion of ssDNA leads to nucleolar disruption.** (A) Lattice-SIM images of dsDNA, ssDNA and NPM1 for semi-intact nuclei without and with S1 nuclease treatment, after treatment with 0.5% Triton X-100 in CSK buffer for 2 min on ice. For the ‘S1 buffer’ sample, semi-intact nuclei were incubated in S1 buffer for 3 h at 37°C. (B,C) Beeswarm plots of mean intensity of NPM1 in nucleoli (C) and nucleoar volume (C). *n*=252 nucleoli from 30 nuclei (S1 buffer), 132 nucleoli from 24 nuclei (S1 nuclease, 1 h), and 84 nucleoli from 16 nuclei (S1 nuclease, 3 h). ****P<0.0001 (two-sided Mann–Whitney *U* test). a.u., arbitrary units.

To investigate the structural roles of ssDNA in nucleolar architecture, we conducted *in situ* digestion of ssDNA in semi-intact nuclei using S1 nuclease (a specific ssDNA endonuclease). ssDNA digestion resulted in the nucleolar disruption with a decrease in the number of hollow patterns seen for NPM1 (Fig. 3A). S1 nuclease treatment decreased the mean intensity of NPM1 in nucleoli (Fig. 3B), indicating leakage or release of NPM1 proteins from the nucleoli, and decreased nucleolar volume (Fig. 3C). Furthermore, even in PFA-fixed semi-intact cells, S1 nuclease treatment resulted in a decrease in the number of hollow patterns of ssDNA and reduction in NPM1 intensity (Fig. S2A), implying that cell signaling did not contribute to this response. We note that the nucleolus has a dynamic structure, which can be affected by biochemical composition, such as Na^+^ ions (Feric et al., 2016; Lafontaine et al., 2021). In our data, the hollow pattern in pre-fixed semi-intact nuclei was not as prominent as shown in Fig. S2A. Thereby, we investigated how the buffers used in this experiment affect nucleolar architecture. We found that the CSK buffer used for semi-intact nuclei transiently attenuates the hollow pattern of NPM1 in 2 min, whereas the nucleolar architecture with hollow patterns of NPM1 was restored in S1 buffer in 10 min (Fig. S2B). This would be the reason why hollow patterns of NPM1 are only not prominent in the pre-fixed semi-intact nuclei, which were fixed immediately after a CSK buffer wash. Nevertheless, our results on the effect of S1 nuclease in the S1 buffer reveal the structural role of ssDNA in nucleolar encapsulation.

### Contribution of ssDNA and histone H1 to nucleolar encapsulation

**Fig. 4.**
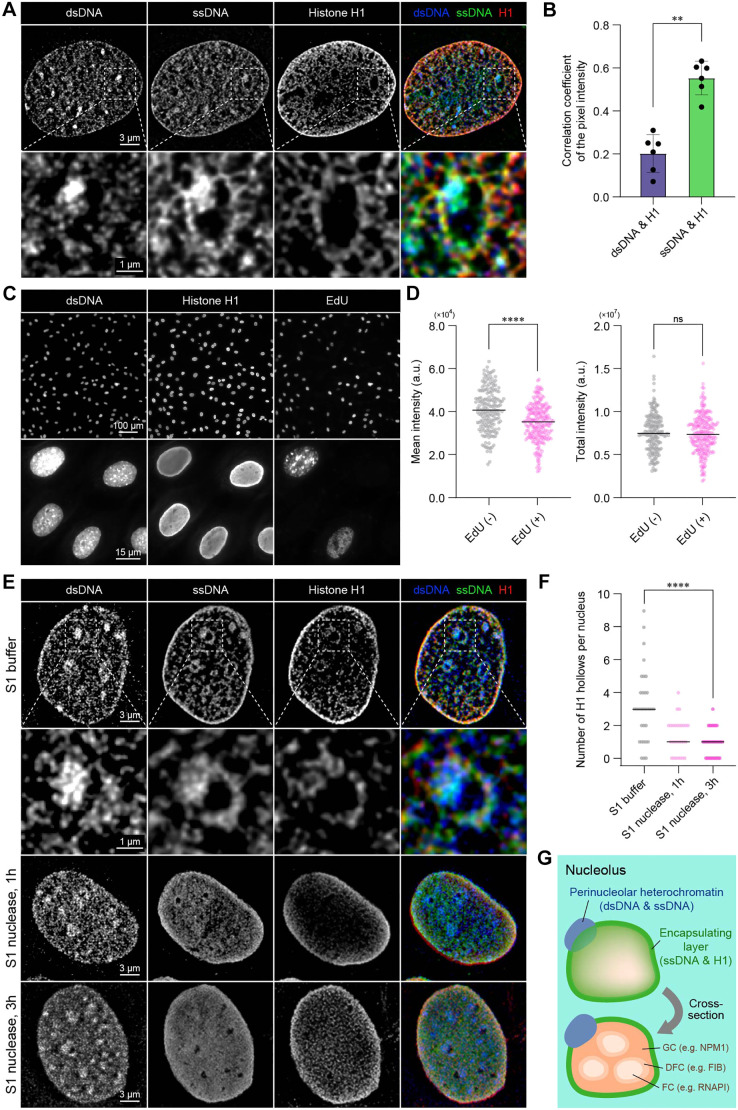
**ssDNA and histone H1 form a molecular complex for nuclear encapsulation.** (A) Lattice-SIM images of dsDNA, ssDNA and histone H1. (B) Pearson correlation coefficient between dsDNA and histone H1, and ssDNA and histone H1. *n*=6 regions with a 5 µm×5 µm range around each nucleolus, from six independent nuclei. **P<0.01 (two-sided Mann–Whitney *U* test). (C) Fluorescence images of dsDNA, histone H1 and EdU. 50 nM EdU was incorporated prior to the fixation and fluorescently detected by Click chemistry. The upper and bottom images were obtained using 20× and 100× objectives, respectively. (D) Beeswarm plots of mean intensity and total intensity of histone H1 for EdU-negative and EdU-positive cells. *n*=186 nuclei (EdU-negative) and 232 nuclei (EdU-positive). ****P<0.0001 (two-sided Mann–Whitney *U* test). (E) Lattice-SIM images of dsDNA, ssDNA and histone H1 for semi-intact nuclei without and with S1 nuclease treatment. The ‘S1 buffer’ sample was incubated in S1 buffer for 3 h at 37°C. (F) Beeswarm plots of the number of hollow patterns of histone H1 per nucleus without and with S1 nuclease treatment. *n*=32 nuclei (S1 buffer), 28 nuclei (S1 nuclease, 1 h), and 34 nuclei for (S1 nuclease, 3 h). ****P<0.0001 (two-sided Mann–Whitney *U* test). (G) Structural schematics of nucleolus encapsulation by a molecular complex of ssDNA and histone H1. a.u., arbitrary units.

To investigate the maintenance of ssDNA outside the nucleolus and its contribution to nucleolar encapsulation, our study explored the molecular complex of ssDNA and histone H1. Although previous findings have indicated the formation of ssDNA and histone H1 condensates in *in vitro* and computational assays (Leicher et al., 2022) as well as the propensity for histone H1 to bind to nucleolar organizer regions (NORs) (Chen et al., 2018), it remains uncertain whether the molecular complex is formed within the nucleus. Lattice-SIM imaging revealed the colocalization of ssDNA and histone H1 outside the nucleolus with hollow-shaped patterns (Fig. 4A). Based on the analysis of the Pearson correlation coefficient of pixel intensity (Fig. 4B), we observed a correlation between the distribution of ssDNA and histone H1, and a relatively weak correlation between dsDNA and histone H1. This finding agrees with previous reports (Kowalski, 2016; Mayor et al., 2015) on the association of histone H1 with the nucleolus. Given that the expression of histone H1 was cell cycle-independent (Fig. 4C,D) and the antibody targets histone variant H1.0, we suggest that ssDNA associates with histone H1.0, a cell-cycle independent histone H1 (Happel and Doenecke, 2009; Kowalski, 2016). *In situ* digestion of ssDNA via S1 nuclease resulted in a decrease in the number of hollow patterns for both ssDNA and histone H1 (Fig. 4E,F). This result indicates that histone H1 associates with ssDNA inside a nucleus. We also observed droplet formation by ssDNA and histone H1 in an *in vitro* reconstitution assay (Fig. S3). These results suggest that the molecular complex of ssDNA and histone H1 plays pivotal roles in nucleolar encapsulation (Fig. 4G).

## DISCUSSION

In this study, we uncovered the mechanism behind the nucleolar encapsulation of ssDNA, a crucial component of the nucleus. Previous research has demonstrated that ssDNA interacts with several proteins to form condensates (Renger et al., 2022), which could contribute to nuclear architecture (Leicher et al., 2022; Nott et al., 2016). However, the lack of observation of ssDNA within the nucleus has impeded further investigation. Using *in situ* imaging, we present direct evidence of ssDNA-based nucleolar encapsulation. The nucleolus self-organizes through phase separation into three distinct layers (FC, DFC, and GC) (Frottin et al., 2019; Leicher et al., 2022). Nevertheless, the impact of external components, such as perinucleolar chromatin, on this process required further research. This study highlights the structural contribution of an ssDNA-based molecular complex to nucleolar encapsulation. In future, detailed analysis of this ssDNA-based molecular complex will be needed to understand, for example, whether it forms a liquid phase inside a nucleus.

The origin of ssDNA in the nucleolus can be partly explained by R-loop formation, which is mediated by RNA polymerase II. To maintain nucleolar organization outside the nucleolus, RNA polymerase II activities are responsible (Panse et al., 1999). The RNA polymerase II-generated R-loop serves as a barrier, inhibiting RNA polymerase I activities at transcription sites of intergenic non-coding RNA (Abraham et al., 2020). The DNA–RNA hybrid and flanking single-stranded DNA present in the R-loop (Santos-Pereira and Aguilera, 2015) might enable ssDNA in the R-loops to form a molecular complex with ssDNA-binding proteins, such as histone H1, RPA (Leicher et al., 2022) and Rad51 (Ding et al., 2023). This has the potential to enclose the nucleolus structurally and regulate actions of RNAPI, which are related to the generation of ribosomes. The ssDNA signals were also prominent at the perinuclear heterochromatic region (as shown in Fig. 2A). R-loop formation could be also the reason for this, given that delayed transcription in heterochromatin produces flanking ssDNA. Thus, the dynamics of R-loop formation in the perinucleolar and perinuclear regions needs to be further investigated.

Although the imaging technique for intranuclear ssDNA was verified in terms of abundance in each nucleus in this study, it is important to address potential limitations and areas for improvement in future investigations. One limitation to consider is the dissociation constants between ssDNA and the dyes used in this study (SYBR Green II^TM^ and Qubit^TM^), which might vary based on nucleotide composition, as previously indicated (Han et al., 2019). Furthermore, certain secondary structures of ssDNA, such as G4 or DNA–RNA hybrids, might impede the attachment of dyes to ssDNA. In addition, even though the size of the dyes used in this research is similar to that of typical DNA staining agents like Hoechst dyes (Saarnio et al., 2020), the accessibility of the dyes to ssDNA might vary based on how tightly packed the heterochromatin is. Researchers should keep these aspects in mind in future studies, depending on their objectives.

In this study, we identified histone H1 (especially the cell cycle-independent H1.0 variant) to be a possible binding partner of the ssDNA that forms the nucleolar encapsulation. Given that histone H1 is known to be involved in high-order chromatin organization (Liu et al., 2023; Ramakrishnan, 1997; Vyas and Brown, 2012; Yusufova et al., 2021), our result suggests a possible link between ssDNA and chromatin organization mediated by histone H1. For example, histone H1 could mediate local chromatin remodeling in response to ssDNA production by genomic processes, such as transcription and replication. Interestingly, a study suggests that histone H1.0 mediates the mechanical behaviors of chromatin structure (Hu et al., 2024). As we observed in lattice-SIM microscopy, ssDNA and histone H1 are accumulated at perinuclear heterochromatin region as well as the perinucleolar region. Transcription at perinuclear heterochromatin could also produce ssDNA and induce chromatin remodeling mediated by histone H1. The contribution of the ssDNA–histone H1 complex to perinuclear heterochromatin needs to be explored in future research.

The production and maintenance of ssDNA inside the nucleus are of broad interest in various research fields. For example, an excess amount of ssDNA in cells is one hallmark of cell death (Kawarada et al., 1998) and senescence (SenGupta et al., 2021), and ssDNA-targeted damage and repair mediated by poly(ADP-ribosyl)ation (Krüger et al., 2020; Zandarashvili et al., 2020) are implicated as fundamental events in the clinical setting. Furthermore, in the fields of biophysics and mechanobiology, dsDNA-ssDNA transition by mechanical forces has been extensively studied using *in vitro* and computational reconstitution experiments at the molecular level and cell biology experiments at the cellular and subcellular levels. However, there have been few bridging points between investigations at the cellular and subcellular levels, in part because of a bottleneck in observing ssDNA inside the cell nucleus. Our *in situ* approach will help us understand how ssDNA is produced and maintained in the cell nucleus under endogenous mechanical forces, such as tensile and torsional forces generated by DNA and RNA polymerases (Fukute et al., 2024), condensin (Terakawa et al., 2017) and myosin VI (de Lanerolle, 2012; Hari-Gupta et al., 2022), as well as exogenous mechanical forces, such as tensile force (Maki et al., 2016, 2018; Nava et al., 2020; Yonemura et al., 2010) and hydrostatic pressure (Maki et al., 2021; Montagne et al., 2017) loaded on a tissue.

## MATERIALS AND METHODS

### Cell culture

The MC3T3-E1 mouse osteoblast-like cells (ATCC) and the mouse embryonic fibroblasts (MEFs; ScienCell Research Laboratories Co.) were cultured in α-MEM (Gibco 32571-036) and DMEM (ATCC 30-2002), respectively, both supplemented with 10% fetal bovine serum (Gibco) and 1% antibiotic-antimycotic (Gibco) (denoted complete medium) in a humidified incubator at 37°C with 5% CO_2_. For fluorescence imaging, 15,000 cells were seeded on a 14-mm-diameter glass bottom dish (p35g-0-14-c, MatTek Co.) 24 h before the experiment. The cell line was routinely checked for contamination by quantitative (q)PCR.

### Fluorescence labeling of ssDNA in cells

Cells were fixed in 4% paraformaldehyde (PFA) in PBS(−) (PBS without Ca^2+^ and Mg^2+^) for 15 min and permeabilized with 0.5% Triton X-100 in PBS(−) for 15 min. Both procedures were performed at room temperature. For an antibody test, methanol fixation (for 5 min at −20°C) was optionally utilized. For RNA digestion, cells were treated with RNase A (1 mg/ml; Nippon Gene Co.) in PBS(−) for 1 h at 37°C. The dsDNA was stained with DAPI (1:500 dilution; 342-07431, FUJIFILM Wako Pure Chemical Co.), which binds to the minor groove of dsDNA. ssDNA and RNA were stained with selective staining reagents SYBR Green II^TM^ (1:5000 dilution; Thermo Fisher Scientific), a widely used cyanine dye (Saarnio et al., 2020), and Qubit^TM^ ssDNA reagent (1:1000 dilution; Thermo Fisher Scientific). The EdU-incorporation assay was conducted with the Click-iT^TM^ Plus EdU Cell Proliferation Kit for Imaging (Thermo Fisher Scientific). Cells were incubated in 50 nM EdU-containing complete medium 3 h prior to fixation.

### Enzymatic digestion and heat denaturation for semi-intact nuclei

Enzymatic digestion of dsDNA and ssDNA in semi-intact nuclei was performed using a modified version of Nickerson's protocol (Nickerson et al., 1997). Cells were first washed with cold PBS(−) and treated with 0.5% Triton X-100 in cytoskeletal (CSK) buffer [10 mM PIPES pH 6.8, 300 mM sucrose, 100 mM NaCl, 3 mM MgCl_2_, 1 mM EGTA and cOmplete™ Proteinase inhibitor and Pierce™ Phosphotase inhibitor] for 2 min at 4°C. After soluble proteins in the cell nuclei were removed by washing with CSK buffer twice, the cells were treated with digestion enzymes for nuclear acids at the following concentrations in each supplied buffer: 500 U/ml DNase I (dsDNA/ssDNA endonuclease; 314-08071, Nippon Gene Co.) and 10 U/ml S1 nuclease (ssDNA endonuclease; 2410A, Takara Bio). The reaction temperature was 37°C. Heat denaturation was performed for 5 min at 90°C on a hot plate. After the enzymatic digestion or heat denaturation, cells were washed with PBS(−) twice and fixed with 4% PFA for 40 min at 4°C. Enzyme-treated samples were subjected to dsDNA, ssDNA and immunofluorescence staining after RNase A treatment (1 mg/ml) in PBS(−) for 1 h at 37°C.

For the Qubit^TM^ assay, semi-intact nuclei after enzymatic digestion or heat denaturation were collected into a microtube by scraping, fixed with 4% PFA for 40 min at 4°C and treated with RNase A (1 mg/ml) in PBS(−) for 1 h at 37°C. In each step, nuclei were washed with PBS(−) by centrifugation (500 ***g*** for 5 min). After the number of nuclei were counted, concentrations of dsDNA and ssDNA in the nuclear suspension were measured with a Qubit 4 Fluorometer^TM^ [Q32851 (dsDNA High-Sensitive) and Q10212 (ssDNA), Thermo Fisher Scientific], in the same manner as in a previous study where nuclear acids on magnetic beads were directly quantified using the Qubit 2.0 fluorometer (Jensen et al., 2013).

### Immunofluorescence staining

Fixed cells were permeabilized with 0.5% Triton X-100 in PBS(−) and blocked with 5% bovine serum albumin (BSA). The samples were incubated for 1 h at room temperature with primary antibody in 1% BSA and 0.03% Triton X-100 in PBS(−), followed by washing in PBS(−), and incubated with secondary antibody in 1% BSA and 0.03% Triton X-100 in PBS(−). Primary antibodies against the following proteins were used: nucleophosmin 1 (NPM1) (1:500 dilution; FC-61991, Thermo Fisher Scientific), fibrillarin (1:500 dilution; #2639, Cell Signaling Technology), RNA polymerase I (RPA194) (1:500 dilution; SC-48385, Santa Cruz Biotechnology) and histone H1 (1:100 dilution; SC-8030, Santa Cruz Biotechnology).

### Microscopy observation and image analysis

All fluorescence images were obtained by epifluorescence microscopy (IX83; Evident Co.) using 20×, 60× or 100× objectives. Images were acquired at room temperature using the same settings (LED power and exposure time) for all samples in each experiment. A lattice-structured illumination microscope (lattice-SIM; Elyra 7, Carl Zeiss Co.) was used for super-resolution microscopy (Fukute et al., 2024) with a Plan-Apochromat 40×/1.4 oil objective lens, four diode laser beams (405, 488 and 561 nm), and an sCMOS camera, using sequential scanning with a step size of 0.2–0.4 μm. Images were analyzed using Fiji software (Schindelin et al., 2012); no custom functions were used in the analysis. For the speckle analysis in the epifluorescence images, all the images were smoothed with background subtraction and binarized with the same threshold to detect speckles inside nucleus. The hollow pattern analysis was similarly conducted for the inverted images where hollows are identified as ‘speckles’. 3D suite (Ollion et al., 2013) was utilized to identify the centroid and the mean intensity and volume of the nucleolus based on NPM1 signals. Then, the intensity profile from the centroid of the nucleolus to *x*-axis and the distance to the point with the maximum NPM1 signal was analyzed.

### Analysis of the correlation coefficient of the pixel intensity

Based on the *Z*-stack lattice-SIM image with multicolor channels, a squared region with a side length of 5 µm (which corresponds to 100×100 pixels in the image) at one focal plane was chosen around each nucleolus and the Pearson correlation coefficient was analyzed (Westin et al., 2014). The Pearson correlation coefficient *r* of the pixel intensity of the two channels for the two markers (*x* and *y*) was calculated using Eqn (1), 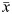 and 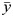 are the mean values of *x* and *y*, respectively. *n*=10,000 pixels:
(1)

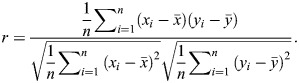


### *In vitro* reconstitution assay for droplet formation by ssDNA and histone H1

ssDNA with the sequence 5′-TTTTTCCTAGAGAGTAGAGCCTGCTTCGTGG-3′, modified with FITC at the 5′ end, was obtained (Thermo Fisher Scientific). This sequence forms droplets with Ddx4n1 (Stender et al., 2021; Nott et al., 2015). For droplet formation, FITC-modified ssDNA was conjugated with histone H1 protein (Sigma-Aldrich) in DNase-free water or 150 mM NaCl (in water), and incubated for 30 min at 37°C.

## Supplementary Material



10.1242/joces.262039_sup1 Supplementary information
