# Supplementary material for: Super-resolution imaging reveals nucleolar encapsulation by single-stranded DNA
Source: J Cell Sci. 2024 Oct 4;137(20):jcs262039. doi: 10.1242/jcs.262039 (PMC11463959; doi:10.1242/jcs.262039)
Supplement: Supplementary information [file joces-137-262039-s1.pdf]

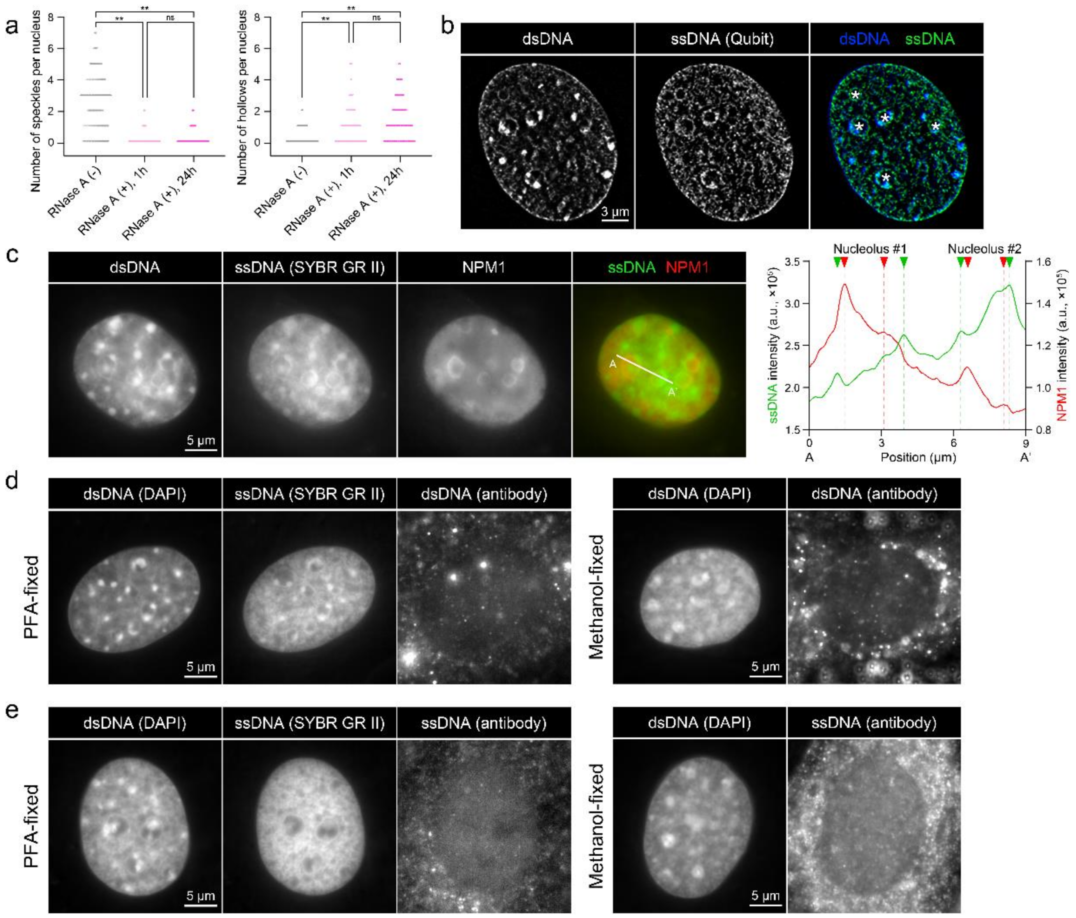

Fig. S1. (a) Beeswarm plots of the number of speckles and hollows per nucleus. Statistical significance was assessed using the Two-sided Steel-Dwass test. $P^{**} < 0.05$. ns, not significant. **(b)** Lattice-SIM images of dsDNA (DAPI) and ssDNA (Qubit™ dye) after RNase A treatment of MC3T3-E1 cells. Hollow-shaped structures (nucleoli) were marked with asterisks in the merged image. **(c)** Fluorescence images of dsDNA (DAPI), ssDNA (SYBR Green II) and NPM1, after RNase A treatment of mouse embryonic fibroblasts. The right panel shows intensity profiles of ssDNA and NPM1. **(d, e)** Fluorescence images of dsDNA (DAPI), ssDNA (SYBR Green II) and immunofluorescence (dsDNA (d) and ssDNA (e), respectively). Paraformaldehyde and methanol fixation were conducted prior to staining procedures.

Journal of Cell Science • Supplementary information

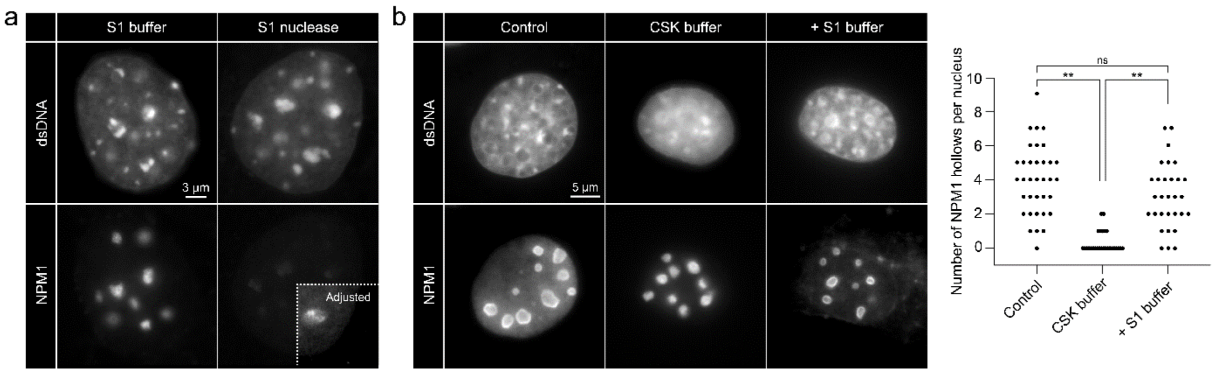

Fig. S2. (a) Fluorescence images of dsDNA and NPM1 without and with S1 nuclease treatment for pre-PFA-fixed semi-intact nuclei treated with 0.5% Triton-X in CSK buffer for 2 min. A contrast-adjusted image is added for the sample with S1 nuclease treatment. **(b)** Fluorescence images of dsDNA (DAPI) and NPM1 for cells just fixed with PFA ("Control"), semi-intact nuclei fixed with PFA ("CSK buffer"), and semi-intact nuclei further treated with S1 buffer for 10 min and fixed with PFA ("+S1 buffer"). The right panel shows a beeswarm plot of the number of hollow patterns of NPM1 per nucleus. n = 248 hollows/35 nuclei ("Control"), 100 hollows/27 nuclei ("CSK buffer"), and 198 hollows/30 nuclei ("+S1 buffer"). Statistical significance was assessed using the Two-sided Steel-Dwass test. $P^{**} < 0.05$. ns, not significant.

Journal of Cell Science • Supplementary information

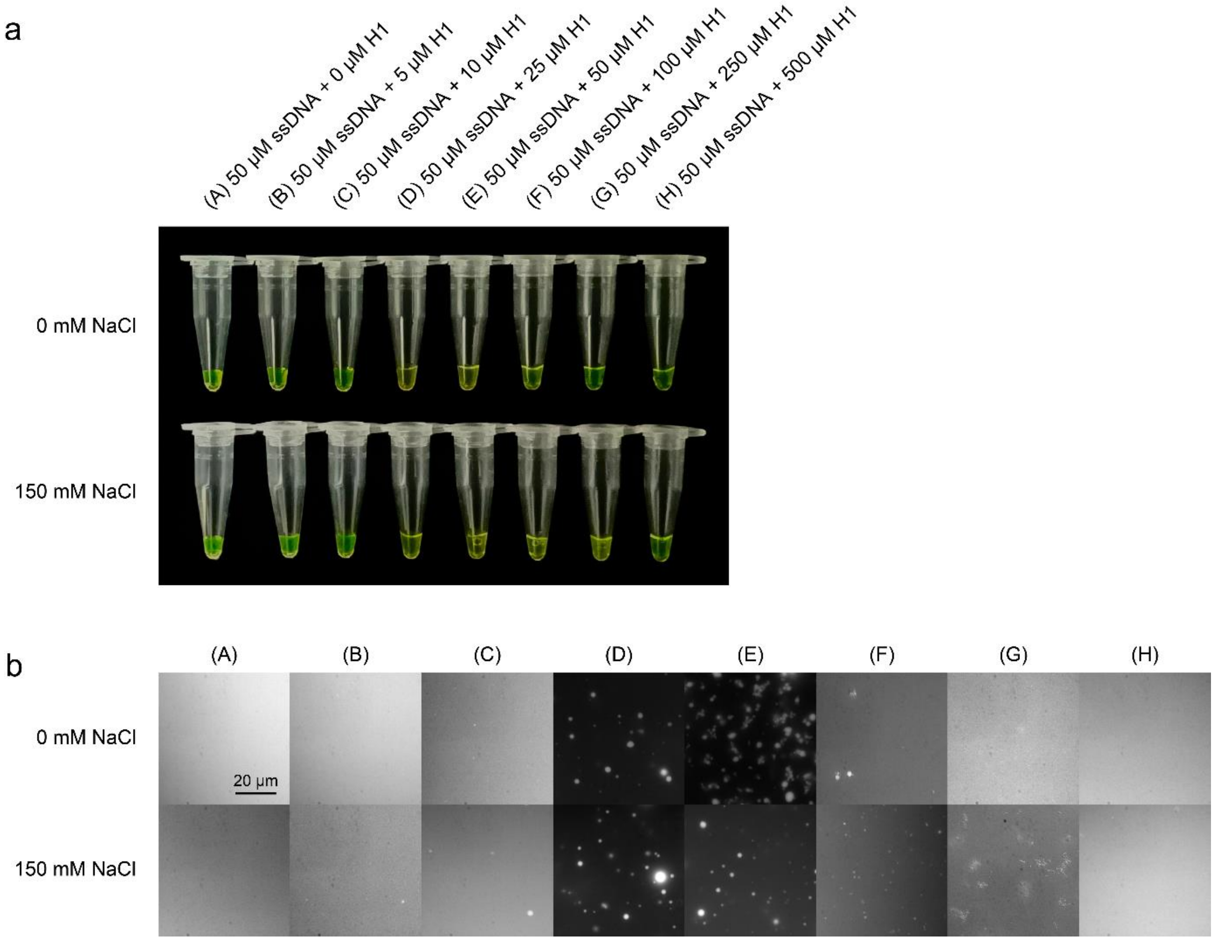

Fig. S3. (a) *In vitro* reconstitution of droplets by FITC-modified ssDNA and histone H1. **(b)** Fluorescence images of FITC in the conditions (A)-(H) without and with 150 mM NaCl in DNase-free water.

Journal of Cell Science • Supplementary information
