# Supplementary material for: Super-resolution imaging reveals nucleolar encapsulation by single-stranded DNA
Source: J Cell Sci. 2024 Oct 4;137(20):jcs262039. doi: 10.1242/jcs.262039 (PMC11463959; doi:10.1242/jcs.262039)
Supplement: Supplementary file 1 [file joces-137-262039_review_history.pdf]

Super-resolution imaging reveals nucleolar encapsulation by single-stranded DNA

Koichiro Maki, Jumpei Fukute and Taiji Adachi
DOI: 10.1242/jcs.262039

Editor: Guillaume Jacquemet

Review timeline

Original submission:	19 February 2024
Editorial decision:	5 April 2024
First revision received:	10 July 2024
Accepted:	15 August 2024

Original submission

First decision letter

MS ID#: JOCES/2024/262039

MS TITLE: Super-resolution imaging reveals nucleolar encapsulation by single stranded DNA

AUTHORS: Koichiro Maki, Jumpei Fukute, and Taiji Adachi

We have now reached a decision on the above manuscript.

To see the reviewers' reports and a copy of this decision letter, please go to: https://submit-jcs.biologists.org and click on the 'Manuscripts with Decisions' queue in the Author Area. (Corresponding author only has access to reviews.)

As you will see, the reviewers raise a number of substantial criticisms that prevent me from accepting the paper at this stage. They suggest, however, that a revised version might prove acceptable, if you can address their concerns. If you think that you can deal satisfactorily with the criticisms on revision, I would be pleased to see a revised manuscript. We would then return it to the reviewers.

Please ensure that you clearly highlight all changes made in the revised manuscript. Please avoid using 'Tracked changes' in Word files as these are lost in PDF conversion.

I should be grateful if you would also provide a point-by-point response detailing how you have dealt with the points raised by the reviewers in the 'Response to Reviewers' box. Please attend to all of the reviewers' comments. If you do not agree with any of their criticisms or suggestions please explain clearly why this is so.

Reviewer 1

Advance summary and potential significance to field

In this study, the authors aim to understand the behaviour and structural roles of single-stranded DNA (ssDNA) within the nucleus, particularly its involvement in nucleolar architecture. Initially, researchers validated using SYBR Green II reagent for in situ fluorescent imaging of ssDNA. Subsequently, super-resolution lattice-structured illumination microscopy (lattice-SIM) was

© 2024. Published by The Company of Biologists under the terms of the Creative Commons Attribution License (https://creativecommons.org/licenses/by/4.0/).

employed to understand the ssDNA distribution. Results revealed hollow patterns of ssDNA within cell nuclei, particularly outside the nucleolus. Co-staining with nucleolar proteins identified regions of ssDNA abundance, predominantly outside the nucleolus.
Furthermore, treatment with S1 nuclease reduced the abundance of NPM1, suggesting leakage of nucleolar proteins, even in PFA-fixed cells. This observation implies a potential structural role of ssDNA in nucleolar encapsulation.
Potential condensate formation between ssDNA and histone H1 was explored to understand the maintenance of ssDNA outside the nucleolus. Lattice-SIM imaging revealed co-localization of ssDNA and histone H1 outside the nucleolus, with a correlation between their distributions. In situ digestion of ssDNA led to the disappearance of hollow patterns for both ssDNA and histone H1, indicating condensate formation. In vitro assays further supported the notion of histone H1 protein condensing ssDNA, suggesting that ssDNA forms condensate with histone H1 to encapsulate the nucleolus.

Comments for the author

While the scientific approach of the study is commendable, however, there are specific questions which must be addressed to support the suggested findings:
1) The authors proposed an in vitro method that uses several dyes, such as SYBR Green II and Qubit dye, to visualise ssDNA. However, the authors also identify the uses as potential limitations of the above study, as addressed in the third paragraph of the Discussion, which is reasonable. In this scenario, would it not be beneficial to use antibodies specific for ssDNA (MAB3299) and dsDNA (MAB1293)?
2) The authors utilised the Qubit assay to quantify dsDNA and ssDNA, as shown in Fig 1B. Still, the materials and method section does not contain sufficient information or the kit used to quantify DNA content in µg.
3) In section 2.2, line 9, the authors quote the intranuclear ssDNA as hollow-shaped and refer to Figure S2. However, please attribute the false colour to dsDNA, ssDNA, NPM1, and its merged version.
4) In section 2.3, the author quotes about the uses of S1 nuclease treatment the disappearance of the hollow pattern, and the reduction of NPM1(Figure S3).
However, the NPM1 signal does not appear hollow here, like in Fig. 2d and Fig. S2.
This discrepancy in the data needs to be explained.
5) In the above study, the authors mentioned the condensation of the H1 with ssDNA on the outer shell of the nucleus. However, what variant of H1 is involved is not clear since the references cited in the study refers to different variants like Reference 17, (Leicher, R. et al.) quoted for H1 co-localisation with ssDNA states about the H1.4, whereas reference 23 (Chen, J. et al.), 24(Kowalski, A), 25(Mayor, R. et al) states about H1.2 and H1.0 respectively.

<u>Reviewer 2</u>

Advance summary and potential significance to field

Maki and colleagues provide a concise study on the role of single stranded DNA (ssDNA) on nucleolar integrity using an elegant combination of super-resolution microscopy imaging, nucleolar markers, RNAse and DNAse for differentiating the contribution of RNA, ssDNA or dsDNA in the patterns observed. The different observation led to the conclusion that the nucleolus of mouse cells is surrounded by a layer of ssDNA contributing nucleolus stability, which is a novel and intriguing finding. Furthermore, linker histones seems tightly associated with this stabilising structure, although its contribution to nucleolus architecture still remains to be investigated.

Comments for the author

The manuscript is well written, concise and well-illustrated particularly featuring informative super resolution microscopy images.

Yet, while the work provides quantifications on several instances, some claims are based on images only. The reader is thus asked indirectly to believe that these are representative, whereas

© 2024. Published by The Company of Biologists under the terms of the Creative Commons Attribution License (https://creativecommons.org/licenses/by/4.0/).

biological variability is very likely to occur. A quantification of the frequency of observation would be appropriate and might reveal more than just the presence / absence of a specific pattern, with some intermediate patterns worth commenting on.

Since these observations surely have been made on more than one sample, the authors are kindly asked to provide their scoring for the experiments claiming
- droplet-like structures using SYBR-Green (Fig.1) with and without RNAse treatment.
- nucleolar disruption using S1 nuclease (Fig.3)
- hollow H1-based structures and their disruption using S1 nuclease (Fig.4)

Similarly, how informative is the H1-ssDNA droplets image? Usually, a dilution series of each components allow to elucidate the stoechiometry or thresholds contributing the LLPS properties. Could such titration experiments be provided in supplementals?

Minor comments
Fig 2 also shows abundant ssDNA signal at the nuclear periphery. Could the authors perhaps comment on this briefly too?

Fig 4 histone H1 distribution is represented as a fiber structure which is misleading. A dotty pattern spreading alongside chromatin fibers also present around the nucleolus, and in close association with ssDNA, might be better suited.

Since H1 is found to be associated with ssDNA, could the authors discuss a bit more the possible role of H1 (discussion), possibly sketching one or several hypotheses?

P1L8 “is encapsulated by single-stranded (ss) DNA-based condensates” and in other parts of the manuscript: can the ssDNA layer coating the nucleolus really be called a “condensate”? That ssDNA forms condensate is not debated, but rather the use of “condensate” here for describing a structure that forms a layer.

Methods- Correlation coefficient of pixel intensities.
Please provide further explanation on this approach which aims at quantifying the colocalization of ssDNA and H1 at the pixel-based level. Does it correspond to a common colocalization approach? And if yes, which one?. Does n represents the number of pixel in a region of interest and i a specific pixel in the image?

Fig 1
1a, please provide also a magnified example for DAPI stained nuclei to compare with the magnified images of SYBR-Green stained nuclei.
1a, please provide a scale bar for the magnified images

First revision

Author response to reviewers' comments

Reviewer 1 Advance Summary and Potential Significance to Field:

In this study, the authors aim to understand the behaviour and structural roles of single-stranded DNA (ssDNA) within the nucleus, particularly its involvement in nucleolar architecture. Initially, researchers validated using SYBR Green II reagent for in situ fluorescent imaging of ssDNA. Subsequently, super-resolution lattice-structured illumination microscopy (lattice-SIM) was employed to understand the ssDNA distribution. Results revealed hollow patterns of ssDNA within cell nuclei, particularly outside the nucleolus. Co-staining with nucleolar proteins identified regions of ssDNA abundance, predominantly outside the nucleolus. Furthermore, treatment with S1 nuclease reduced the abundance of NPM1, suggesting leakage of nucleolar proteins, even in PFA-fixed cells. This observation implies a potential structural role of ssDNA in nucleolar encapsulation. Potential condensate formation between ssDNA and histone H1 was explored to understand the

© 2024. Published by The Company of Biologists under the terms of the Creative Commons Attribution License (https://creativecommons.org/licenses/by/4.0/).

maintenance of ssDNA outside the nucleolus. Lattice-SIM imaging revealed co-localization of ssDNA and histone H1 outside the nucleolus, with a correlation between their distributions. In situ digestion of ssDNA led to the disappearance of hollow patterns for both ssDNA and histone H1, indicating condensate formation. In vitro assays further supported the notion of histone H1 protein condensing ssDNA, suggesting that ssDNA forms condensate with histone H1 to encapsulate the nucleolus.

We are grateful to the reviewer for the constructive comments that have helped us improve the manuscript. All comments have been carefully considered and appropriate changes have been made in the revised manuscript. The response to each individual comment is given in blue font.

Reviewer 1 Comments for the Author:

While the scientific approach of the study is commendable, however, there are specific questions which must be addressed to support the suggested findings:

(a) The authors proposed an in vitro method that uses several dyes, such as SYBR Green II and Qubit dye, to visualise ssDNA. However, the authors also identify the uses as potential limitations of the above study, as addressed in the third paragraph of the Discussion, which is reasonable. In this scenario, would it not be beneficial to use antibodies specific for ssDNA (MAB3299) and dsDNA (MAB1293)?

We appreciate the author's suggestion to use antibodies for ssDNA (MAB3299) and dsDNA (MAB1293). Although we wished to utilize the antibodies for further verification, the immunofluorescence staining with the antibodies resulted in quite low, cytoplasmic signals in both PFA and Methanol fixation (the new Supp. Figs. S1d and S1e). Especially for ssDNA antibody (MAB3299), the manufacture states that apoptotic cells can be identified with this antibody. For dsDNA antibody (MAB1293), previous literatures (Wu et al., Theranostics, 2020; Lam et al., Cancer Res., 2014) reported cytoplasmic signals only after treatment with H_2O_2 and Ara-C, which induce DNA damage. Such information suggests that those antibodies can only target cytoplasmic, damaged/fragmented DNA upon genotoxic events.

We have added **Supp. Figs. S1d and S2e** and the corresponding parts in **Results (Page (P.) 6, Line (L.) 9-12)**.

(b) The authors utilised the Qubit assay to quantify dsDNA and ssDNA, as shown in Fig 1B. Still, the materials and method section does not contain sufficient information or the kit used to quantify DNA content in µg.

We apologize for not providing the detailed information for Qubit assay.

We have added the experimental procedure of Qubit assay in **Methods (P. 14, L. 18 - P. 15, L. 6)**.

(c) In section 2.2, line 9, the authors quote the intranuclear ssDNA as hollow-shaped and refer to Figure S2. However, please attribute the false colour to dsDNA, ssDNA, NPM1, and its merged version.

We have amended **Supp. Fig. S1b** with a merged version and arrows indicating nucleoli.

(d) In section 2.3, the author quotes about the uses of S1 nuclease treatment, the disappearance of the hollow pattern, and the reduction of NPM1(Figure S3). However, the NPM1 signal does not appear hollow here, like in Fig. 2d and Fig. S2. This discrepancy in the data needs to be explained.

We appreciate the critical comment by the reviewer, and apologize for the low-resolution image in the previous Supp. Fig. S3 (the new Supp. Fig. S2a). Indeed, the NPM1 staining only in this data did not show a clear hollow pattern, but a filled pattern with holes. We tried to understand the reason and found that the transient treatment with cytoskeletal (CSK) buffer for permeabilization results in decrease in the hollow patterns of NPM1, which was restored further treatment with S1 buffer.

We have added **Supp. Fig. S2b** and the corresponding parts in **Results (P. 7, L. 9-18)**.

(e) In the above study, the authors mentioned the condensation of the H1 with ssDNA on the outer shell of the nucleus. However, what variant of H1 is involved is not clear since the references cited in the study refers to different variants like Reference 17, (Leicher, R. et al.) quoted for H1 co-

© 2024. Published by The Company of Biologists under the terms of the Creative Commons Attribution License (https://creativecommons.org/licenses/by/4.0/).

localisation with ssDNA states about the H1.4, whereas reference 23 (Chen, J. et al.), 24(Kowalski, A), 25(Mayor, R. et al) states about H1.2 and H1.0 respectively.

We thank to the reviewer's the professional comment. The antibody used in this study targets histone H1.0 (H1f0), which is cell cycle-independent, and therefore we did not observe higher fluorescence intensity in EdU-positive (DNA synthesizing) than in negative cells. Since the contribution by each histone isoform remains unclear, we would like to focus on discussing the role of histone H1.0 in nucleolar architecture.

We have added **Figs. 4c and 4d** and the corresponding parts in **Results (P. 8, L. 12-14) and Discussion (P. 10, L. 16 - P. 11, L. 7).**

*****

Reviewer 2 Advance Summary and Potential Significance to Field:

Maki and colleagues provide a concise study on the role of single stranded DNA (ssDNA) on nucleolar integrity using an elegant combination of super-resolution microscopy imaging, nucleolar markers, RNAse and DNAse for differentiating the contribution of RNA, ssDNA or dsDNA in the patterns observed. The different observation led to the conclusion that the nucleolus of mouse cells is surrounded by a layer of ssDNA contributing nucleolus stability, which is a novel and intriguing finding. Furthermore, linker histones seems tightly associated with this stabilising structure, although its contribution to nucleolus architecture still remains to be investigated.

We thank to the reviewer for the positive assessment of our study and suggestions for the improvement. The response to individual comments is given in blue font.

Reviewer 2 Comments for the Author:

The manuscript is well written, concise and well-illustrated particularly featuring informative super resolution microscopy images.

(a) Yet, while the work provides quantifications on several instances, some claims are based on images only. The reader is thus asked indirectly to believe that these are representative, whereas biological variability is very likely to occur. A quantification of the frequency of observation would be appropriate and might reveal more than just the presence / absence of a specific pattern, with some intermediate patterns worth commenting on. Since these observations surely have been made on more than one sample, the authors are kindly asked to provide their scoring for the experiments claiming
-droplet-like structures using SYBR-Green (Fig.1) with and without RNAse treatment.
-nucleolar disruption using S1 nuclease (Fig.3)
-hollow H1-based structures and their disruption using S1 nuclease (Fig.4)

Thank you very much for the reviewer's professional comment. We have performed the population analysis for the following data, for additionally obtained lattice-SIM images:

- Figs. 1b and 1c: The number of speckles and hollows per nucleus in SYBR GRII images
- Figs. 3b and 3c: Mean intensity of NPM1 in nucleoli and nucleolar volume
- Fig. 4f: The number of H1 hollows per nucleus in histone H1 images

We have added **Figs. 1b-c, 3a-c, 4e-4f** and the corresponding parts in **Results (P. 5, L. 8-10, P. 7, L. 4-7, and P. 8, L. 14-16).**

(b) Similarly, how informative is the H1-ssDNA droplets image? Usually, a dilution series of each components allow to elucidate the stoechiometry or thresholds contributing the LLPS properties. Could such titration experiments be provided in supplementals?

We agree with the reviewer for important comment. We have checked that lipid droplet formation by ssDNA and histone H1 occurs at the equivalent molar ratio, while it is still unknown whether the molecular complex forms liquid phase inside a nucleus. This data should be included as a supplementary material.

We have added **Supp. Fig. S3** and the corresponding parts in **Results (P. 8, L. 16-18)** and **Discussion (P. 9, L. 10-12).**

Minor comments

(c) Fig 2 also shows abundant ssDNA signal at the nuclear periphery. Could the authors perhaps comment on this briefly too?

Thank you very much for the comment to broaden the scope of our manuscript. Indeed, ssDNA signal at the perinuclear region would be worth to discuss.

© 2024. Published by The Company of Biologists under the terms of the Creative Commons Attribution License (https://creativecommons.org/licenses/by/4.0/).

We have added the corresponding explanations in **Results (P. 6, L. 8-9)** and **Discussion (P. 11, L. 3-7)**.

(d) Fig 4 histone H1 distribution is represented as a fiber structure which is misleading. A dotty pattern spreading alongside chromatin fibers also present around the nucleolus, and in close association with ssDNA, might be better suited.

We appreciate the reviewer's constructive comment. We have amended **Fig. 4g**, by simply illustrating the encapsulating layer by ssDNA and histone H1.

(e) Since H1 is found to be associated with ssDNA, could the authors discuss a bit more the possible role of H1 (discussion), possibly sketching one or several hypotheses?

Thank you again for the positive assessment. We have added the corresponding explanations in **Discussion (P. 10, L. 16 - P. 11, L. 7)**.

(f) P1L8 "is encapsulated by single-stranded (ss) DNA-based condensates" and in other parts of the manuscript: can the ssDNA layer coating the nucleolus really be called a "condensate"? That ssDNA forms condensate is not debated, but rather the use of "condensate" here for describing a structure that forms a layer.

We agree with the reviewer's comment. The expression of "condensates" based on our results was inappropriate - we could not probe that the molecular complex by ssDNA and histone H1 forms phase-separated condensates or droplets (in liquid phase).

We carefully corrected all the expression related to our results in the entire manuscript.

(g) Methods- Correlation coefficient of pixel intensities. Please provide further explanation on this approach which aims at quantifying the colocalization of ssDNA and H1 at the pixel-based level. Does it correspond to a common colocalization approach? And if yes, which one?. Does n represents the number of pixel in a region of interest and i a specific pixel in the image?

We apologize for the unclear explanation in the analysis of correlation coefficient. In this analysis, we focused on a squared region with a side length of 5 μm (=100 pixels) around the nucleolus and evaluated the Pearson correlation coefficient of fluorescent intensity between blue (dsDNA), green (ssDNA) and red (histone H1) channels, by referring to the reference [59]. n represents the total number of pixels (10,000 pixels) in each region, and i represents a specific pixel.

We have amended the corresponding parts in **Methods (P. 16, L. 17 - P. 17, L. 3)**.

(h) Fig 1 1a, please provide also a magnified example for DAPI stained nuclei to compare with the magnified images of SYBR-Green stained nuclei.

Thank you very much. We have added the magnified image for DAPI staining in **Fig. 1a**.

Second decision letter

MS ID#: JOCES/2024/262039

MS TITLE: Super-resolution imaging reveals nucleolar encapsulation by single-stranded DNA

AUTHORS: Koichiro Maki, Jumpei Fukute, and Taiji Adachi

I am happy to tell you that your manuscript has been accepted for publication in Journal of Cell Science, pending standard publication integrity checks.

Reviewer 1

Advance summary and potential significance to field

The manuscript uses super-resolution microscopy to contribute to our understanding of the behaviour and structural roles of single-stranded DNA within the nucleus, particularly its involvement in nucleolar architecture.

© 2024. Published by The Company of Biologists under the terms of the Creative Commons Attribution License (https://creativecommons.org/licenses/by/4.0/).

Comments for the author

The authors have addressed most of my criticisms and improved the original manuscript. I endorse the revised manuscript.

Reviewer 2

Advance summary and potential significance to field

The authors have done very good revisions which fully satisfy my requests.

Comments for the author

The authors have done very good revisions which fully satisfy my requests.

Only a minor comment for Fig1 b-c. The Y axis of the graph bears the legend “probability (%)” – why probability? If correct, indicate the calculation method.
Or is it simply the % of cells (nuclei)?

© 2024. Published by The Company of Biologists under the terms of the Creative Commons Attribution License (https://creativecommons.org/licenses/by/4.0/).
